# Dietary Curcumin Alleviated Acute Ileum Damage of Ducks (*Anas platyrhynchos*) Induced by AFB1 through Regulating Nrf2-ARE and NF-κB Signaling Pathways

**DOI:** 10.3390/foods10061370

**Published:** 2021-06-14

**Authors:** Sanjun Jin, Hao Yang, Yihan Jiao, Qian Pang, Yingjie Wang, Min Wang, Anshan Shan, Xingjun Feng

**Affiliations:** Laboratory of Molecular Nutrition, Institute of Animal Nutrition, Northeast Agricultural University, Changjiang Street 600#, Xiangfang District, Harbin 150030, China; Sanjunjin@163.com (S.J.); yanghao951209@163.com (H.Y.); yihanjiao11@163.com (Y.J.); pangqian1210@163.com (Q.P.); 18846091206@163.com (Y.W.); 17854221802@163.com (M.W.); asshan@neau.edu.cn (A.S.)

**Keywords:** curcumin, acute ileum, AFB1-DNA adducts, Nrf2-ARE, ducks

## Abstract

Aflatoxin B1 (AFB1) is a stable toxic metabolite threatening health of human and animal and widely contaminated animal feed and human food. This present study aimed to investigate the effects of dietary curcumin on ileum injury in ducks induced by AFB1 administration and explore its underlying mechanisms. Ducks (*N* = 450, one-day-old male) with a similar weight were randomly assigned to 3 groups, containing the control group, AFB1 group (60 μg AFB1 kg^−1^ body weight) and curcumin (500 mg curcumin kg^−1^ diet) + AFB1 group. AFB1 administration markedly increased the ileum damage, AFB1-DNA adducts in the plasma and oxidation stress and inflammation. Adding curcumin into diet protected the ileum against morphology damage induced by AFB1 administration, decreased AFB1-DNA adducts in the plasma and eliminated oxidation stress and inflammation in the ileum of ducks. Anti-oxidation and anti-inflammatory effects of curcumin could protect the ileum against acute damage via activating Nrf2-ARE signaling pathway and inhibiting NF-κB signaling pathway. Conclusively, curcumin was a dietary anti-oxidation and anti-inflammation agent via activating Nrf2-ARE signaling pathway and inhibiting NF-κB signaling pathway to protect ileum against acute damage induced by AFB1 administration.

## 1. Introduction

Meat is an important source of high-quality protein for human nutrition. Duck meat is abundantly consumed worldwide, especially in Asia because of its desirable nutritional characteristics [1]. Therefore, the breeding of ducks has attracted people’s attention. For ducks, there are various disadvantages in the breeding process, such as AFB1 that threaten the health of ducks. Aflatoxin B1 (AFB1) is one of table toxic metabolites produced by *Aspergillus species*. AFB1 is recognized as the most toxic among aflatoxin (AF) groups, along with an assortment of toxic effects to threat the health of human and animals [2]. For people or animals, food or feed is a common and important way to exposure to AFB1, but inhalation and direct contact with skin or mucosa contact are also counted and not ignored [3]. Previous studies have proved that AFB1 exerts a potent toxicity that is very complex and strong, resulting in growth retardation, biological malformations, liver toxicity, digestive tract disorders and even cancer [4,5]. AFB1 obtained from food or mucosa contact had negative effects on respiratory systems, digestive system and tissues and growth performance [3,6,7]. Tissue and organ damages induced by AFB1 administration related to oxidation stress and inflammation. AFB1 administration marked increased AFB1-DNA adducts content in injured organ [8]. The absorption and conversion of nutrients and toxins were occurred in the stomach and small intestine, so the small intestine mucosal immune system is the first line to protect bodies against injury [9]. The functionality and morphology of the health gastrointestinal tract could be destroyed by unfavorable factors such as toxicants, bacteria and viruses [10,11]. AFB1 induced the destruction of the intestinal structure, manifested by shedding of epithelial cells in jejunal villus and lymphocytic cell infiltration in the intestine of chicken [12,13]. The variation of functionality and morphology may be attributed to the process of toxin metabolism that is often accompanied by oxidative stress and inflammation. It is an urgent matter to find an antidote to reduce and minimize the threat of AFB1 for people and animals. Consequently, AFB1 is one of the foremost concerns in poultry industry due to its potent toxicity.

Curcumin is a kind of polyphenol component occurring in turmeric rhizomes (*Curcuma Longa Linn*) as a functional feed additive used in livestock feed [14], and widely used as a green and natural spice, colorant, preservative and flavoring in the food industry. Plant extracts such as curcumin containing polyphenols improved the body’s immunity [15]. Curcumin plays a key role against oxidative stress mediated pathological conditions and it has anti-inflammatory activities when used as a therapy for treatment and prevention of chronic diseases [16,17]. The clinical trials indicated that curcumin have not severe toxic or side effect, and with anti-carcinogenic and anti-toxicogenic properties, which led to curcumin as an attractive chemopreventive agent against AFB1-induced tissues damage [18,19,20]. Literatures demonstrated that curcumin has ability to anti-inflammation, anti-apoptosis and antioxidation by activating the Nrf2/HO-1 pathway, upregulated antioxidation capacity to eliminate inflammation and oxidative stress in the body and eliminate AFB1-induced oxidative stress [21,22,23,24,25]. Curcumin suppressed oxidation stress and inflammasome formation by activating Nrf2-ARE and inhibiting NF-κB signaling pathway in remnant kidney [26], demonstrating anti-oxidation and anti-inflammatory property. In addition, dietary curcumin significantly improved the antioxidant capacity in broilers [27] and ducks [28]. There was no attempt to correlate the role of curcumin in Nrf2-ARE and NF-κB signaling pathway and in animal model of acute AFB1 administration. This study shed light on this issue and provided a theoretical basis for curcumin as a feed additive to protect duck’s health against AFB1 administration and reduce economic losses of feed and breeding industries caused by AFB1 pollution.

## 2. Materials and Methods

### 2.1. Chemicals

Curcumin was purchased from Nanjing Nutri-herb Biotech Co., Ltd. (Nanjing, Jiangsu, China, CAS: 458-37-7), its purity was more than 98% by HPLC analysis. AFB1 (purity ≥ 98%, CAS NO. 1162-65-8) was purchased from Shanghai Yuan ye Bio-Technology Co., Ltd. (Shanghai, China). Antibodies used in this study were purchased from Beyotime Biotechnology, Shanghai, China, including GAPDH (Catalog number: AG019), Nrf2 (Catalog number: AF7623), Phospho-NF-κB p65 (Ser276) (Catalog number: AF5875), HRP-labeled Goat Anti-Rabbit IgG (H + L) (Catalog number: A0208) and HRP-labeled Goat Anti-Mouse IgG (H + L) (Catalog number: A0216).

### 2.2. Ducks and Husbandry

The experimental protocol was conducted in accordance with the practices outlined in the Guide for the Care and Use of Agricultural Animals in Agriculture Research and Teaching of Northeast Agricultural University (Protocol number: NEAU-[2011]-9).

Ducks (*n* = 450, one-day-male *Anas platyrhynchos*, 33.8 ± 0.2 g) with no significant different weight were purchased from a commercial hatchery and randomly assigned to 3 groups (Table 1), with 10 replicate pens (cages) per group and 15 ducks per pen for a 70-day feeding trial. The basal diets were formulated according to National Research Council (1994). Ducks in the T_0_ and T_0_ + AFB1 group were fed a corn-soybean basal diet (Table 2), ducks in the T_500_ + AFB1 group were fed the basal diet supplemented with 500 mg of curcumin kg^−1^ of diet (T_500_) a 70-day trial. On the 70 days, ducks with similar body weight in the T_0_ + AFB1 and T_500_ + AFB1 groups were fed 60 μg of AFB1 kg^−1^ of body weight, and ducks in the T_0_ group were fed the equal volume of PBS solution. Ducks were fed in Acheng Experimental Base of Northeast Agricultural University and provided with ad libitum access to water and powdered diets. Fifteen ducks with similar body weight (1.4 ± 0.3 kg) from each group were selected and fasted for 12 h, then fed PBS solution to ducks in T_0_ group and 60 μg of AFB1 kg^−1^ of body weight to ducks in T_0_ + AFB1 and T_500_ + AFB1 groups at same time. After 12 h, the fifteen ducks in each group were to obtain duck samples. 

### 2.3. Sample Collection

Blood samples (10 mL) were obtained using heparin tubes from veins of duck wings and centrifuged at 1000× *g* for 15 min at 4 °C. The obtained plasma was immediately separated and stored at −80 °C for analyzing. Ducks were anesthetized by inhaling ether and killed to obtain ileum. The ileum was washed 3 times in PBS, immediately and individually stored in a liquid nitrogen tank then at −80 °C for qRT-PCR and antioxidant capacity analysis. Then, about 0.125 cm^3^ ileum was obtained and put into 4% paraformaldehyde solution for tissues section and about 1 mm^3^ ileum were put into electron microscopic solution at 4 °C for later ultrastructural observation.

### 2.4. Assay of Antioxidant Levels in the Plasma

Plasma levels of total superoxide dismutase (T-SOD), glutathione peroxidase (GSH-Px), Glutathione S-transferase (GSH-ST) and malondialdehyde (MDA) were measured by assay kits (Nanjing Jiancheng Bioengineering Institute, Nanjing, China), respectively, with UV-VIS Spectrophotometer (UV1100, MAPADA, Shanghai, China). 

### 2.5. Assay of AFB1-DNA Adducts Levels in the Plasma

The generation of AFB1-DNA adducts in the plasma were determined using ELISA kits according to the kit’s specifications (Nanjing Jiancheng Bioengineering Institute, Nanjing, China).

### 2.6. Assay of Antioxidant Ability in Ileum

Ileum (100.00 mg) was devolved and mixed in 0.9 mL stroke-physiological saline solution (4 °C, 0.9% NaCl, pH = 7.2–7.4) to obtain 10% ileum/SPSS homogenate. The activity or content of total superoxide dismutase (T-SOD U/mg Protein), reductive Glutathione (GSH-PX μmol/mg Protein), Glutathione S-transferase (GSH-ST U/mg Protein) and Malondialdehyde (MDA nmol/mg Protein) in ileum were assessed using assay kits (Nanjing Jiancheng Bioengineering Institute, Nanjing, China), respectively, with a UV-VIS spectrophotometer (UV1100, MAPADA, Shanghai, China). 

### 2.7. RNA Isolation and Real-Time Quantitative Polymerase Chain Reaction (qRT-PCR)

Total RNA of the duck ileum (100.00 mg) was isolated using a reagent kit (TaKaRa, Japan) according to the protocol recommended by manufacturers. The concentration and purity of total RNA were detected at A260/A280 ratio with a spectrophotometer (IMPLEN, Germany). 1 μg total RNA in each sample was converted into the cDNA with a Prime Script™ RT reagent kit with gDNA Eraser (TaKaRa, Dalian, China) according to the protocol recommended by manufacturers. The obtained cDNA from each duck ileum was used as a template for a TB Green™ Premix Ex Taq™ (TaKaRa, Dalian, China) RT-PCR (qRT-PCR) kit. The gene accession number of ducks was obtained from NCBI and the duck gene primers were synthesized by Sangon Biotech Co., Ltd. (Shanghai, China) (Table 3). The relevant gene expression in the duck ileum was determined by the Quanta gene Q225 thermal cycler apparatus. The qRT-PCR was run in Monad Selected Real-Time PCR System (ABI 7500 real-time PCR instrument (USA)) flowing to the condition: one cycle at 95 °C for 30 s, 40 cycles at 95 °C for 5 s and at 60 °C for 30 s. The relative gene expression ratio of detection mRNA was detected using the 2^−ΔΔCt^ method and normalized to β-actin expression. 

### 2.8. Western Blotting

The duck ileum was pulverized and lysed in RIPA buffer containing 1 mmol/L PMSF (Beyotime, Shanghai, China) in the ice. Total protein concentration of the ileum was determined by a bicinchoninic acid (BCA) assay kit (Nanjing Jiancheng Bioengineering Institute, Nanjing, China). 12% and 10% SDS-polyacrylamide gel for electrophoresis were used to obtain target proteins with different molecular weight. Then, target proteins were transferred to a polyvinylidene-difluoride (PVDF) membrane (Beyotime, Shanghai, China) for blots with a transblotting apparatus. The PVDF membrane was washed 3 times for 10 min each time in 1 × PBST and then blocked 2 h in 5% skim milk. The PVDF membrane was washed 3 times again, and incubated with GAPDH, Nrf2 and P-P65 (Beyotime Biotechnology, Shanghai, China) primary antibodies for 8–12 h at 4 °C, respectively. Next day, the PVDF membrane was washed 3 times again, and incubated with corresponding horseradish peroxidase labeled antibody at 37 °C for 1 h, then washed 3 times again. Target protein bands were detected and visualized under the action of the enhanced fluorescence detection kit BeyoECL Star (Beyotime Biotechnology, Shanghai, China). Images of blots were recorded and analyzed by the Essential V6 imaging platform (UVITEC, Cambridge, England). GAPDH protein served as an internal control protein. All the results of experiment were repeated in triplicate. The relative expressions of target proteins were expressed as the ratio of band intensities of proteins to GAPDH. 

### 2.9. Statistical Analysis

The experiment data were obtained by at least six times and each sample was measured three times. Analysis of the research data using Independent-Samples T Test by SPSS (Version 22.0, SPSS Inc., Chicago, IL, USA) with 5% probability of error and statistical significance was *p* < 0.05 in this study.

## 3. Results

### 3.1. Intestinal Morphology

**Figure 1:**
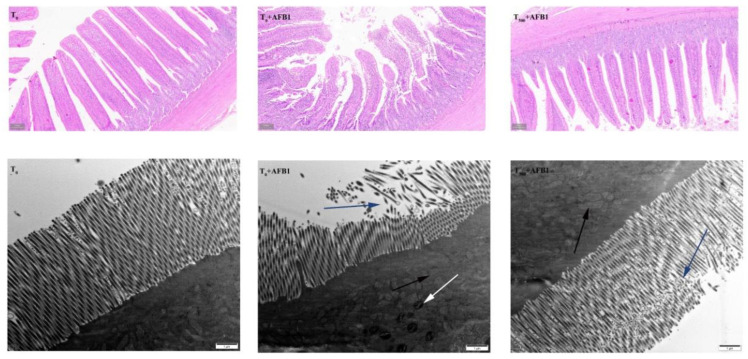
Ileum histopathological examination and scanning electron microscope of ducks (*Anas PlatyrhynchosI*) exposed to AFB1 at 70 days. The black arrowhead indicated swollen mitochondria, the white arrowhead indicated the shrinkage of mitochondrial and the blue arrowhead indicated broken of intestinal microvilli. T_0_: the control group, T_0_ + AFB1: AFB1 group; T_500_ + AFB1: curcumin + AFB1 group.

In this study, histopathological examination (H&E) and the destruction of the microstructure assessed by an ultrastructural of ileum had been demonstrated in Figure 1. Compared to the T_0_ group, the ileum in the T_0_ + AFB1 group showed the epithelial thickness reduction, the villi structure damage and the inflammatory cell aggregation and the microstructure destruction, such as a large number of microvilli severely broken and mitochondria swelled and shrinkage. The damage in ileum of ducks containing structural destruction of villi and microvilli after AFB1 administration induced the appearance of inflammation and oxidation stress. As expected, dietary curcumin had an ability to protect the ileum against acute damage induced by AFB1 administration, including few of ileum villi broken, inflammatory cell gathered and a little damage of ileum structure evaluated by H&E, and decreasing the number of broken microvilli, reducing mitochondrial swelling and eliminating mitochondrial shrinkage assessed by an ultrastructural as shown in structure difference between the T_500_ + AFB1 group and the T_0_ + AFB1 group.

### 3.2. Levels of AFB1-DNA Adducts in the Plasma

**Figure 2:**
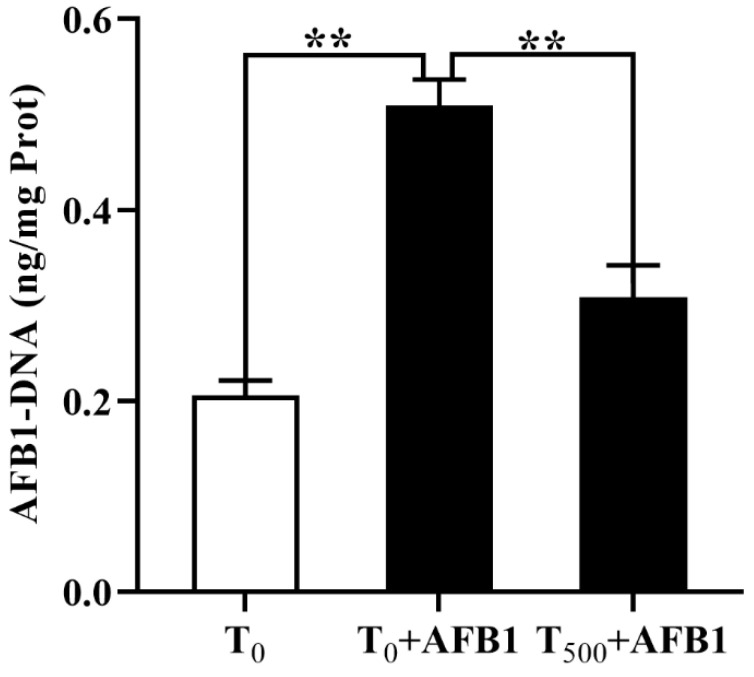
The aggregation of AFB1-DNA adducts in the plasma of ducks (*Anas PlatyrhynchosI*) exposed to AFB1 at 70 days. T_0_: the control group, T_0_ + AFB1: AFB1 group; T_500_ + AFB1: curcumin + AFB1 group. Values are expressed as Mean ± SEM (*n* = 15), ** means *p* < 0.01.

AFB1-DNA adducts in the plasma of ducks was measured by indirect competitive ELISA and shown in Figure 2. Compared to the T_0_ group, AFB1 administration significantly increased the content of AFB1-DNA adducts (*p* < 0.001) in the plasma. As expected, dietary curcumin reduced AFB1-DNA adducts content (*p* = 0.001) in the plasma of ducks in the T500 + AFB1 group relative to that in the T_0_ + AFB1 group. 

### 3.3. Antioxidant Capacity in the Plasma and Ileum

**Figure 3:**
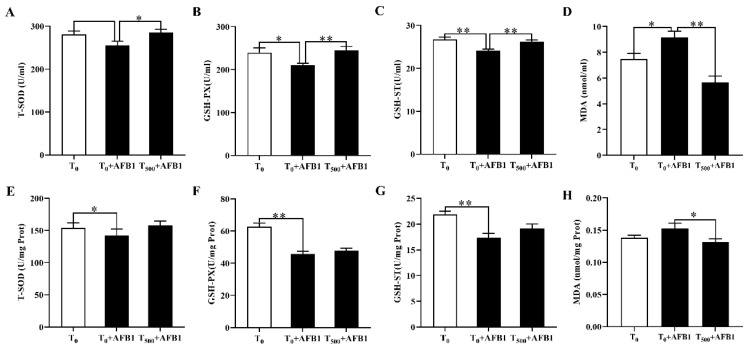
Antioxidant capacity of the plasma and ileum of ducks (*Anas PlatyrhynchosI*) exposed to AFB1 at 70 days. T_0_: the control group, T_0_ + AFB1: AFB1 group; T_500_ + AFB1: curcumin + AFB1 group. T-SOD, Total Superoxide Dismutase; GSH-PX, Glutathione Peroxidase; GSH-ST, Glutathione S—transferase; MDA, Malondialdehyde. (**A**–**D**) means antioxidant capacity in the plasma, (**E**–**H**) means antioxidant capacity in the ileum. Values are expressed as Mean ± SEM (*n* = 15), and * means *p* < 0.05, ** means *p* < 0.01.

The antioxidant capacity of the plasma was shown in Figure 3. Exposure of AFB1 led to oxidation stress, manifesting that the T-SOD (*p* = 0.073), GSH-PX (*p* = 0.034) and GSH-ST (*p* = 0.003) activities in the plasma were decreased in the T_0_ + AFB1 group than those in the T_0_ group. However, the T-SOD (*p* = 0.039), GSH-PX (*p* = 0.009) and GSH-ST (*p* = 0.003) activities were significantly enhanced in the T_500_ + AFB1 group than those in the T_0_ + AFB1 group. The concentration of MDA (*p* = 0.028) in the plasma was increased in the T_0_ + AFB1 group than that in the T_0_ group, and the concentration of MDA (*p* < 0.001) in the plasma was decreased in the T_500_ + AFB1 group than those in the T_0_ + AFB1. 

### 3.4. Expression of Genes Related to Nrf2-ARE Signaling Pathway in the Ileum

**Figure 4:**
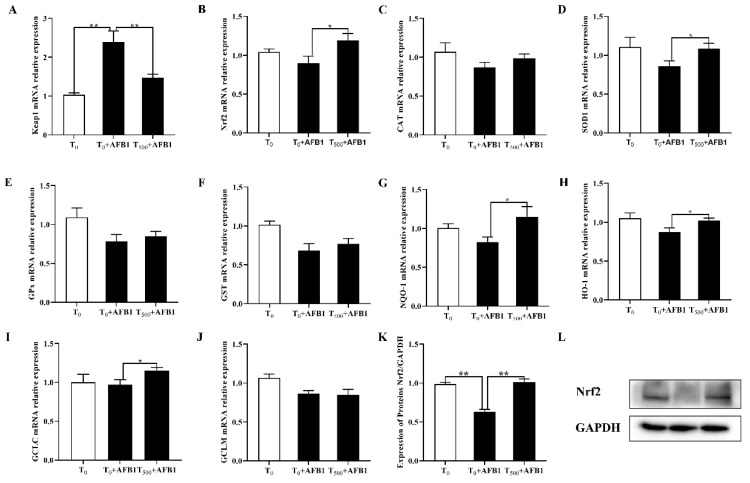
mRNA and protein expression of genes related to Nrf2-ARE signaling pathway in the ileum of ducks (*Anas PlatyrhynchosI*) exposed to AFB1 at 70 days. T_0_: the control group, T_0_ + AFB1: AFB1 group; T_500_ + AFB1: curcumin + AFB1 group. Keap1, Kelch-like ECH-associated protein (**A**); Nrf2, Nuclear factor erythroid 2-related factor 2 (**B**); CAT, Catalase (**C**); SOD, Superoxide dismutase (**D**); GPx, Glutathione peroxidase (E); GST, Glutathione S-transferase (**F**); NQO1, NAD(P)H quinone oxidoreductase 1 (**G**); HO-1, Heme oxygenase 1 (**H**); GCLC, Glutamate cysteine ligase catalyzes subunits (**I**); GCLM, Glutamic acid cysteine ligase modified subunit (**G**); GAPDH, Glyceraldehyde-3-phosphate dehydrogenase (**K**). Genes including the enzymatic antioxidant system (CAT, SOD1, GPX and GST) and phase Ⅱ detoxification enzymes (NQO-1, HO-1, GCLC and GCLM). The relative expression of Nrf2 protein in the ileum were expressed as the ratio of band intensity of the target protein to internal reference (GAPDH) (**K**,**L**). Values were expressed as Mean ± SEM (*n* = 15), and * means *p* < 0.05, ** means *p* < 0.01.

AFB1 administration induced the cell oxidation stress and further resulted in the expression changes of genes including Keap1, Nrf2, CAT, SOD1, GPX, GST, NQO-1, HO-1, GCLC and GCLM in the ileum of ducks. As shown in Figure 4, AFB1 administration significantly increased the mRNA (*p =* 0.001) level of Keap1 gene, and inhibited mRNA levels of genes including Nrf2 (*p =* 0.171), CAT (*p =* 0.166), SOD1 (*p =* 0.121), GPX (*p =* 0.065), GST (*p =* 0.008), NQO-1 (*p =* 0.061), HO-1 (*p =* 0.068), GCLC (*p =* 0.800) and GCLM (*p =* 0.090) and Nrf2 protein content (*p =* 0.001) in the ileum of ducks in the T_0_ + AFB1 group relative to those in the T_0_ group. As expected, adding curcumin into diet fed ducks for 70 days significantly decreased the mRNA (*p =* 0.012) expression of Keap1 gene in ileum, significantly increased mRNA level of genes including Nrf2 (*p =* 0.042), SOD1 (*p =* 0.038), HO-1 (*p =* 0.041), NQO-1 (*p =* 0.047) and GCLC (*p =* 0.043), and improved mRNA level of genes including CAT (*p =* 0.229), GPX (*p =* 0.568), GST (*p =* 0.454) and GCLM (*p =* 0.860), and Nrf2 protein (*p =* 0.005) in ileum of ducks in the T_500_ + AFB1 group relative to those in the T_0_ + AFB1 group.

### 3.5. Expression of Genes Related to NF-κB Signaling Pathway in the Ileum

**Figure 5:**
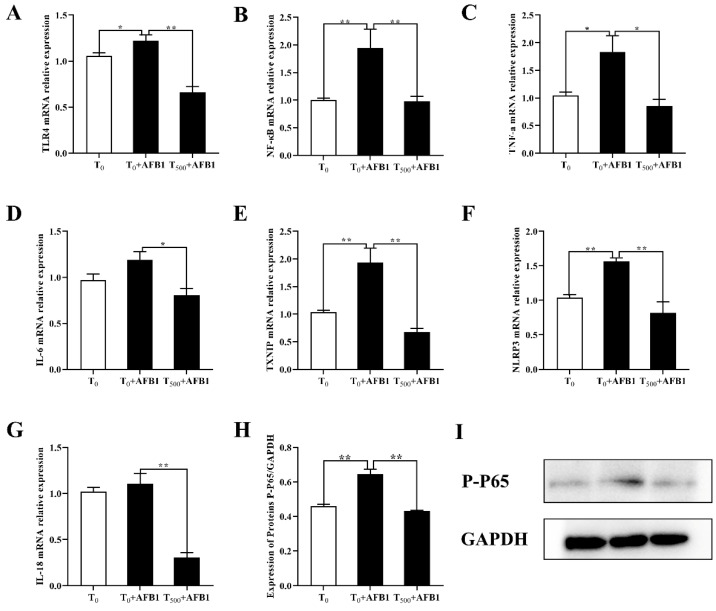
mRNA and protein levels of genes related to NF-κB signaling pathway in the ileum of ducks (*Anas PlatyrhynchosI*) exposed to AFB1 at 70 days. T_0_: the control group, T_0_ + AFB1: AFB1 group; T_500_ + AFB1: curcumin + AFB1 group. TLR4, toll like reporter 4 (**A**); NF-kB, Nuclear factor kB (**B**); TNF-α, Tumor Necrosis Factor-α (**C**); Il-6, Interleukin -6 (**D**); TXNIP, thioredoxin interacting protein (**E**); NLRP3, NOD-like receptor family pyrin domain containing protein 3 (**F**); Il-18, Interleukin -18; P-P 65 (**G**), Phospho-NF-κB p65 (Ser276) rabbit polyclonal antibody (**H**); (**I**) GAPDH, Glyceraldehyde-3-phosphate dehydrogenase. The relative expression of P-P65 protein in the ileum was expressed as the ratio of band intensitiy of the target protein to the internal reference (GAPDH). Values were expressed as Mean ± SEM (*n* = 15), and * means *p* < 0.05, ** means *p* < 0.01.

The expression of inflammatory genes was shown in the Figure 5. Compared to the T_0_ group, AFB1 administration increased mRNA level of some genes such as TLR4 (*p =* 0.037), NF-κB (*p <* 0.001), TNF-α (*p =* 0.025), IL-6 (*p =* 0.072), TXNIP (*p =* 0.007), NLRP3 (*p <* 0.001) and IL-18 (*p =* 0.478) in the ileum of ducks in the T_0_ + AFB1 group. As expected, dietary curcumin significantly suppressed over-expression of genes including TLR4 (*p <* 0.001), NF-κB (*p =* 0.001), TNF-α (*p =* 0.012), IL-6 (*p =* 0.007), TXNIP (*p =* 0.001), NLRP3 (*p =* 0.001) and IL-18 (*p <* 0.001) in the ileum of ducks in the T_500_ + AFB1 group relative to those in the T_0_ + AFB1 group. In addition, the contents of P-P 65 (*p =* 0.004) in the ileum increased in the T_0_ + AFB1 group relative to that in the T_0_ group. As expected, dietary curcumin significantly suppressed P-P 65 (*p =* 0.015) protein content. 

## 4. Discussion

Intestinal morphology is one of behavioral markers to evaluate inflamation and oxidation stress of intestine induced by AFB1 administration. Literatures on the effects of AFB1 adnimistration on ileum morphology of ducks are scantly. Yan et al. (2020) reported that AFB1 administration led to cardiac pathologic damage of Sprague-Dawley rats, inflammatory cell infiltration and greater cardiomyocyte degeneration [29]. Catarrhal enteritis with inflammatory cell infiltrations in the intestine of broiler chickens induced by AFB1 destroyed the structure of intestine [12]. Luzi et al. (2002) reported acute AFB1 administration induced the ileum contractions [30]. The results in this study demonstrated that dietary curcumin is a potent protective agent of ileum agaisnt inhibiting inflamation, which may be that curcumin had an ability to inhibite anti-inflammatory in multiple inflammatory disordies in mice [31,32,33]. 

DNA damage caused by oxidative stress will destruct the stability of DNA, which can promote the formation of various DNA adducts [34]. AFB1-DNA adducts is a biomarker to evaluate the injury degree of body which was induced by AFB1. Synthesizing and enriching of AFB1-DNA adducts destroyed the structure of tissues, then resulting in carcinogenic development [35]. AFB1 would be metabolized by cytochrome P450s isoenzymes to AFB1-8,9-epoxide (AFBO) and produce related adducts [36] and increase tissues damage, oxidative stress and DNA damage by ROS [37]. AFB1-DNA adducts can bound with the nucleoproteins and nucleic acids, thus induce DNA and cell damages and decrease the level of antioxidant enzymes and the protein synthesis [38]. Zhang et al. (2016) reported that curcumin-supplemented inhibited liver damage induced by AFB1 by increasing antioxidation activity of antioxidant enzymes (GPx, SOD, CAT and GST) and inhibiting AFB1-DNA production [39]. In this study, AFB1 administration increased AFB1-DNA adducts content in the plasma. Dietary curcumin significantly diminished this phenomenon, results demonstrated that dietary curcumin was potential to protect ileum in this acute AFB1 administration model that may be explained by the antioxidant effect of curcumin that improved the antioxidation capacity of body [40,41].

Oxidation stress occurred when the imbalance of oxidation and antioxidation in bodies induced by the decreases of antioxidant enzyme activities and the increases in lipid peroxidation levels. Antioxidant enzyme system including CAT, GSH-Px and SOD is the first line of cell defenses against free radicals and reactive oxygen species (ROS) and is indispensable in the entire defense strategy of antioxidants in the body [42]. GST is a crucial enzyme to downregulate reactive oxygen species (ROS) and oxidative stress in order to achieve detoxification for bodies [43]. As shown in Figure 3, oxidation stress in plasma and ileum of ducks occurred during acute ileum damage induced by AFB1 administration. As expected, dietary curcumin ameliorated oxidation stress of bodies by increasing T-SOD, GSH-PX and GSH-ST activities in the plasma and ileum after AFB1 administration. The results in this study demonstrated that the curcumin is a potent anti-oxidation agent for ducks fed AFB1 administration that may be explained by the anti-oxidative capacity via suppressing lipid oxidation and increasing antioxidation enzyme activity by curcumin-supplemented [8,44,45]. These results are in line with a previous report that demonstrated that curcumin ameliorated AFB1-induced alteration in glutathione, SOD, CAT and MDA activities [8]. This may be due to the ability of curcumin to scavenge free radicals by restoring antioxidant enzymes activities and alleviated oxidative stress [15,46].

The balance between oxidation and anti-oxidation in vivo were regulated by T-SOD and GSH-Px [47]. Nrf2 was translocated into the cell nucleus and combined with t antioxidant response element (ARE) and upregulated the transcription of the antioxidant enzyme genes including SOD, CAT, GSH-Px, HO-1, NQO-1, GCLC and GCLM [47,48]. In addition, GST is upregulated by activating Nrf2 signaling way and as a kind of phase-II detoxifying enzyme involved in various detoxification in vivo to relieve oxidative stress [49,50]. Oxidative stress and lipid peroxidation were biomarkers for rats induced by AFB1 administration [51,52]. Oxidative stress was diminished by activation genes expression under Nrf2-ARE signaling pathway, such as NQO-1, HO-1 and GCLC [40]. Numerous research reported that curcumin-supplemented induced genes expression including HO-1, NQO-1, γ-GCLC, γ-GCLM, CAT and GPX via Nrf2 activation in broiler and rats [40,41] and eliminated liver damage induced by AFB1 administration [39,53]. The results in this study indicated that AFB1 administration may induce oxidation stress damage of ileum via inhibiting Nrf2 signaling pathway. Dietary curcumin promoted Nrf2 downstream genes expression such as antioxidant genes (CAT, SOD1, GPX1, GST) and phase Ⅱ detoxifying enzyme genes (NQO1, HO-1, GCLC, GCLM), which demonstrated that adding curcumin into diet for ducks inhibited the acute oxidation damage of ileum induced by AFB1 administration through activating Nrf2-ARE signaling pathway Results in this study provided an evidence that dietary curcumin could be a potent ameliorating agent to protect ileum against oxidation stress induced by AFB1 administration.

Oxidation stress activated NF-κB signaling pathway then further evaluated the production of inflammatory cytokines [54]. In this study, ileum injury induced by AFB1 administration may be due to the inflammation. AFB1 administration may induce ileum damage directly via increasing expression of inflammatory factors, and directly activating the inflammatory signaling pathway. There is a positive relationship between the oxidation stress and the inflammation in tissues [55]. Ko et al. (2020) reported that oxidation stress activated NF-κB signaling pathway, upregulated pro-inflammatory cytokines and caused inflammation in rat lung [56]. The NF-κB signaling could be activated by AFB1 in the cell line 3D4/21 [57], resulting a series of inflammatory reactions [58]. Kumara et al. (2020) found that AFB1 administration induced the inflammatory by elevating levels of pro-inflammatory factors cytokines, TNF-α, IL-12 and IL-6 in the serum of albino mice [59]. Dietary AFB1 exposure resulted in genes over-expression of TNF-α, IL-1β and IL-6 in the liver of pigs, and the mRNA levels of inflammatory factors (TNF-α, IL-12, IL-6) in the liver of pigs fed the diet containing 8% grape seed meal and AFB1 returned to the control levels [60]. The levels of TNF-α, IL-6 and IL-1β in the liver of rats with intra-uterine growth retardation were increased, which in the liver of IUGR rats fed with curcumin 400 mg kg^−1^ diet returned to the control level of normal birth weight rats [38]. The activation of NF-κB signaling pathway upregulated genes expression (NLRP3 and Caspase-1), which promoted IL-1β and IL-18 maturation and secretion and triggered an inflammatory response [61]. Results in this study demonstrated that AFB1 administration significantly evaluated gene expression of inflammation factors in the ileum of ducks, and dietary curcumin inhibited these gene expression, in line with a study that the activation of NLRP3 signaling pathway by AFB1 in rats [40]. Previous results showed that curcumin had an ability to inhibit NLRP3 protein expression via suppressing caspase-1 and IL-1β [29]. Thus, results in this study revealed that curcumin may be one of the promising feed additives to relieve inflammation in ileum and ileum damage induced by AFB1 administration by inhibiting NF-κB signaling pathway.

## 5. Conclusions

**Figure 6:**
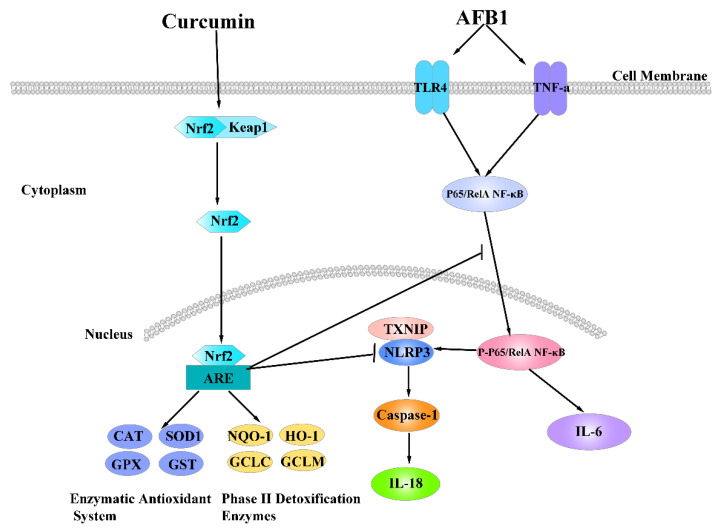
The antioxidant damage mechanism of dietary curcumin through Nrf2-ARE signaling pathway to elevate the expression of its downstream genes, including the antioxidant enzymatic system (CAT, SOD1, GPX and GST) and phase Ⅱ detoxification enzymes (NQO-1, HO-1, GCLC and GCLM), as well as anti-inflammatory mechanism via inhibiting expression of genes related to NF-κB signaling pathway.

In conclusion, AFB1 administration induced ileum injury, oxidation stress and inflammation via inhibiting expression of downstream genes of Nrf2-ARE signaling pathway and activating genes expression of downstream genes of NF-κB signaling pathway. However, dietary curcumin markedly ameliorated ileum damage, oxidation stress and inflammation of ducks induced by AFB1 administration, possible due to activate Nrf2-ARE and inhibit NF-κB signaling pathway (Figure 6). Results in this study provided a powerful evident that dietary curcumin is an effective feed additive to protect the ileum against acute injury induced by AFB1 administration via activating Nrf2-ARE and inhibiting NF-κB signaling pathway.

## Figures and Tables

**Table 1:** Experiment design.

Groups	Basal Diet	Curcumin (mg Curcumin/kg Basal Diet)
T_0_	corn-soybean	0
T_0_	corn-soybean	0 + AFB1
T_500_	corn-soybean	500 + AFB1

T_0_: ducks fed basal diet for 70 days then fed PBS water on 70 day; T_0_ + AFB1: ducks fed basal diet for 70 days then fed 60 μg of AFB1/kg of duck body weight on 70 day; T_500_ + AFB1: ducks fed 500 mg of curcumin kg^−1^ of basal diet for 70 days then fed 60 μg of AFB1 kg^−1^ of duck body weight on 70 day.

**Table 2:** Ingredient composition and nutrient content of the basal diet (%, as-fed basis).

Items	1–4 Weeks ^1^	5–8 Weeks ^2^	9–10 Weeks ^3^
Ingredient			
Corn (7.9)	61.70	68.94	75.80
Soybean meal (45)	26.09	26.80	20.10
Corn protein flour (55)	7.90	-	-
Dicalcium phosphate	1.40	1.40	1.40
Limestone	1.08	1.06	1.06
Salt	0.38	0.38	0.38
DL-Methionine	0.15	0.22	0.16
L-Lysine	0.20	0.10	0.00
choline chloride (50%)	0.10	0.10	0.10
Premix	1.00	1.00	1.00
Total	100	100	100
Nutritional level			
Calculated nutrient ^4^			
Metabolizable energy (MJ/kg)	12.14	11.98	12.21
CP (%)	20.67	17.51	15.03
Calcium (%)	0.90	0.90	0.88
Total phosphorus (%)	0.68	0.67	0.65
Non-phytate phosphorus (%)	0.44	0.44	0.44
Lysine (%)	1.07	0.95	0.71
Methionine (%)	0.48	0.48	0.39
Methionine + cystine (%)	0.81	0.75	0.63
Threonine (%)	0.75	0.66	0.56
Tryptophane (%)	0.21	0.19	0.16

^1^ The premix provided per kilogram diet: vitamin A 4000 IU, vitamin D3 2000 IU, vitamin E 20 mg, vitamin K3 2.0 mg, vitamin B1 2.0 mg, vitamin B2 12 mg, vitamin B6 3.0 mg, vitamin B12 0.02 mg, nicotinic acid 50 mg, D-pantothenic acid 10 mg, folic acid 1 mg, biotin 0.2 mg, Cu 8 mg, Fe 60 mg, Mn 100 mg, Zn 60 mg, Se 0.2 mg, I 0.4 mg. ^2^ The premix provided per kilogram diet: vitamin A 3000 IU, vitamin D3 2000 IU, vitamin E 10 mg, vitamin K3 2.0 mg, vitamin B1 1.5 mg, vitamin B2 8 mg, nicotinic acid 30 mg, D-pantothenic acid 10 mg, vitamin B6 3.0 mg, vitamin B12 0.02 mg, biotin 0.1 mg, folic acid 1 mg, Cu 8 mg, Fe 60 mg, Mn 80 mg, Zn 40 mg, Se 0.2 mg, I 0.4 mg. ^3^ The premix provided per kilogram diet: vitamin A 2500 IU, vitamin D3 1000 IU, vitamin E 10 mg, vitamin K3 2.0 mg, vitamin B1 1.5 mg, vitamin B2 8 mg, nicotinic acid 30 mg, D-pantothenic acid 10 mg, vitamin B6 3.0 mg, vitamin B12 0.02 mg, biotin 0.1 mg, folic acid 1 mg, Cu 8 mg, Fe 60 mg, Mn 80 mg, Zn 40 mg, Se 0.2 mg, I 0.3 mg. ^4^ Values were calculated based on the data provided by Feed Database in China (2004).

**Table 3:** Sequences, product sizes and TM values of primers for target genes.

Transcripts	Accession Number		Gene Sequence (5′–3′)	Product Length (bp)
Keap1	MF774811.1	Forward	TCACCCTCCATAAACCCACCCAAG	102
		Reverse	AGTAGCCCAAGGACTGCCGATAG	102
Nrf2	NM_001310777.1	Forward	GTTGAATCATCTGCCTGTGG	171
		Reverse	TAAGCTAGGTGGTCGAGTGC	172
HO-1	KU048806.1	Forward	AAGAGCCAGGAGAACGGTCACC	139
		Reverse	TGCCCACCAGGTCTGTCTGAC	139
SOD1	XM_013097859.1	Forward	CCTGTGGTGTCATCGGAATA	116
		Reverse	TTGAACGAGGAAGAGCAAGTA	127
GCLC	XM_027455104.1	Forward	TTCAGGTGACATTCCAGGCTTGC	108
		Reverse	AGAACGGAGATGCAGCACTCAATG	108
GCLM	XM_027462629.1	Forward	TGTTGTGTGATGCCACCTGATCTC	150
		Reverse	CCATTCGTGTGCTTTGACGTTCTG	150
CAT	KU048802.1	Forward	TGTGCGTGACTGACAACCAAGG	96
		Reverse	ACATGCGGCTCTCCTTCACAAC	96
NQO-1	XM_027466610.1	Forward	CGTCGCCGAGCAGAAGAAGATC	195
		Reverse	CTGGTGGTGAACGACAGCATGG	195
GST	LOC101797566	Forward	ACAAGGCTGCAACCAGATACTTCC	178
		Reverse	ACTGCACATCTGCTCTGCTAAGC	178
GPX	XM_027459004.1	Forward	GAACGGCACCAACGAGGAGATC	99
		Reverse	TTCACCTGGCACTTCTGGAACAG	99
NLRP3	MH373356.1	Forward	CGCTGAACGAGGACGCACTG	124
		Reverse	TGGAAGGGTAGTCGGGACATAGC	124
TXNIP	XM_032204531.1	Forward	GCTGCCAAGAAGGAGAAGAAGGTG	130
		Reverse	TGTTCTCGAAGTCGGCGTTGATG	130
Caspase-1	XM_027446016.1	Forward	GCGGAACCAAGAGCAGAGATGAG	130
		Reverse	CCACGGCAGGACTGGATAATAACC	130
IL-18	NM_001310420.1	Forward	GGCTCTGTCCCAAGGCAGGAG	124
		Reverse	GCCACTCTGCGTCAGCTTCAC	124
IL-6	JQ728554.1	Forward	ATGTGCGAGAAGTTCACCGTCTG	113
		Reverse	TCGTCGAAGCCAGCCAGGAG	113
TNF-a	XM_013105371.3	Forward	AGCTGGCTAAGACCGTGGTCAG	151
		Reverse	ACGTTGTTGACTCGTCCATGTGAC	151
TLR4	NM_001310413.1	Forward	GACCTCCAGCACACGAAGTTAGAC	165
		Reverse	GGAGTTGCCTGCCATCTTGAGC	165
NF-κB	XM_027453277.2	Forward	GGAGCAGTGGCGGTGTCAAC	126
		Reverse	AGTGCAGTTCATGTCATCGGTCTC	126
β-actin	EF667345.1	Forward	ATGTCGCCCTGGATTTCG	62
		Reverse	CACAGGACTCCATACCCAAGAA	62

## Data Availability

The raw data presented in this study are available on request from the corresponding author.
